# Curcumin- and Piperine-Loaded Emulsomes as Combinational Treatment Approach Enhance the Anticancer Activity of Curcumin on HCT116 Colorectal Cancer Model

**DOI:** 10.3389/fbioe.2020.00050

**Published:** 2020-02-11

**Authors:** Zeynep Busra Bolat, Zeynep Islek, Bilun Nas Demir, Elif Nur Yilmaz, Fikrettin Sahin, Mehmet Hikmet Ucisik

**Affiliations:** ^1^Department of Genetics and Bioengineering, Faculty of Engineering, Yeditepe University, Istanbul, Turkey; ^2^Graduate School of Engineering and Natural Sciences, Istanbul Medipol University, Istanbul, Turkey; ^3^Regenerative and Restorative Medicine Research Center (REMER), Istanbul Medipol University, Istanbul, Turkey; ^4^Department of Biomedical Engineering, School of Engineering and Natural Sciences, Istanbul Medipol University, Istanbul, Turkey

**Keywords:** curcumin, piperine, emulsome, colorectal cancer, combination chemotherapy

## Abstract

Combination chemotherapy, administrating two chemotherapeutic agents concurrently, comes into prominence, as the heterogeneity or the level of the disease necessitates a collaborative action. Curcumin, isolated from turmeric, and piperine, isolated from black long pepper, are two dietary polyphenols studied for their intrinsic anti-cancer properties against various cancer types including colorectal cancer (CRC). Furthermore, piperine improves the therapeutic effect of curcumin. Addressing this mutual behavior, this study combines curcumin and piperine within emulsome nanoformulations. Curcumin- (CurcuEmulsomes) and piperine-loaded emulsomes (PiperineEmulsomes) have established a uniform, stable, spherical dispersion with average diameters of 184.21 and 248.76 nm, respectively. The solid tripalmitin inner core achieved encapsulation capacities of up to 0.10 mg/ml curcumin and 0.09 mg/ml piperine content. While piperine treatment alone – in its both free and emulsome forms – showed no inhibition in the proliferation of HCT116 cells *in vitro*, its presence as the second drug agent enhanced curcumin’s effect. Combination of 7 μM PiperineEmulsome and 25 μM CurcuEmulsome concentrations was found to be most effective with an inhibition of cell proliferation of about 50% viability. Cell cycle arrest at G2/M phase and induced apoptosis verified the improved anti-cancer characteristics of the therapy. While CurcuEmulsomes achieved a fourfold increase in Caspase 3 level, combination of treatment with PiperineEulsomes achieved a sixfold increase in the level of this apoptotic marker. Combinational treatment of HCT116 cells with CurcuEmulsomes and PiperineEmulsomes improved the anticancer activity of the compounds and highlighted the potential of the approach for further *in vivo* studies.

## Introduction

Colorectal cancer is a heterogeneous disease occurring in the colon and the rectum. CRC is the second most commonly diagnosed cancer in females and the third in males with a 1.84 million cases in 2018 (Bray et al., 2018). It accounts for the one of the highest cancer mortality rate, corresponding to 883,200 number of deaths, worldwide (Torre et al., 2015). According to the stage of the cancer and the degree of the complication, current treatment methods are mainly dependent on success of chemotherapy either alone or combined with surgical resection or radiation therapy (Mishra et al., 2013). Diet is also an important factor associated to CRC risk and disease prevention. Indicating the importance of diet in occurrence of the disease, studies show lower incidence of CRC linked with vegetarian diets when compared to carnivore diets (i.e., eating only meat) (Aran et al., 2016). Epidemiological studies revealed that CRC death rate is possible to reduce as much as 90% with correct dietary manipulations benefiting the anti-cancer and anti-oxidant characteristics of natural biocompounds (Goel et al., 2001). In some of these studies, curcumin is very intensively studied on various CRC models for its distinctive biological properties (Johnson and Mukhtar, 2007; Mishra et al., 2013; Yallapu et al., 2013).

Curcumin, chemically known as diferuloyl methane, is a hydrophobic polyphenol, naturally obtained from the rhizome of the plant Curcuma longa (turmeric) (Kunnumakkara et al., 2017; Nelson et al., 2017). With its multifaceted metabolic actions including anti-oxidant and anti-inflammatory activities, curcumin can selectively kill tumor cells (Ravindran et al., 2009; Gupta et al., 2013; Ghosh et al., 2015; Shanmugam et al., 2015; Hewlings and Kalman, 2017). Several clinical trials classify curcumin as a potential chemopreventive and chemotherapeutic agent (Hatcher et al., 2008; Doello et al., 2018). Curcumin has antioxidant effects via activation of the Nuclear factor erythroid 2-related factor 2 (Nrf2) expression (Balogun et al., 2003), and provides protection against oxidative damage induced by ferric nitrilotriacetate (Fe-NTA) (Iqbal et al., 2009). Taking an anti-inflammatory action, curcumin has the ability to inhibit cyclooxygenase-2 (COX-2), lipoxygenase (LOX), and inducible nitric oxide synthase (iNOS), which are essential for inflammatory processes (Menon and Sudheer, 2007; Ghosh et al., 2015; Shanmugam et al., 2015). Again indicating an anti-inflammatory effect, Janus kinase-signal transducer and activator of transcription signaling pathways were downregulated in presence of curcumin (Teiten et al., 2010). Moreover, inhibition of NF-Kβ signaling pathway supports anti-inflammatory effect of curcumin in cancer cells (Singh and Aggarwal, 1995). Curcumin upregulates the heat shock family genes in addition to genes involved in cell cycle (Teiten et al., 2009). Curcumin inhibits angionenesis via inhibition of signal transduction pathways such as AP-1, NF-Kβ and protein kinase C, and shows anti-metastatic activity (Bhandarkar and Arbiser, 2007).

Combination therapy approaches, where a secondary active drug agent or drug candidate is co-administered with curcumin, appear to further benefit from the anti-cancer effect of curcumin. Behaving synergistically, the second agent enhances the curcumin’s anti-cancer activity. Doxorubicin (Zhao et al., 2015; Lv et al., 2016; Dash and Konkimalla, 2017a); docetaxel (Bayet-Robert et al., 2010; Yan et al., 2016); gemcitabine (Dhillon et al., 2008; Epelbaum et al., 2010; Kanai et al., 2011), celebrex (Lev-Ari et al., 2005; Abdallah et al., 2018), paclitaxel (Ganta and Amiji, 2009; Boztas et al., 2013; Abouzeid et al., 2014; Sarisozen et al., 2014; Kim et al., 2016; Yang et al., 2017; Calaf et al., 2018), campthotecin (Xiao et al., 2015), cisplatin (Li et al., 2017), resveratrol (Du et al., 2013), epigallocatechin gallate (EGCG) (Vivar-Quintana et al., 2006; Jin et al., 2017), sulforaphane (Danafar et al., 2018) and piperine (Shaikh et al., 2009; Kakarala et al., 2010; Patial et al., 2015; Vecchione et al., 2016; Tang et al., 2017; Baspinar et al., 2018) are among the active molecules studied in combination with curcumin for their synergistic anti-cancer behavior.

Among the proposed secondary agents, piperine, a dietary polyphenol isolated from black and long peppers, distinguished with its intrinsic features, does not only improve curcumin’s existing anti-cancer activity, but also its extremely poor bioavailability (Teiten et al., 2010; Patial et al., 2015; Tang et al., 2017). Besides, piperine alone possesses anti-mutagenic and anti-tumor influences (Srinivasan, 2007; Chinta et al., 2015). Piperine has been widely reported to inhibit the growth of colon cancer cell lines by G1 arrest in cell cycle and by triggering apoptosis (Yaffe et al., 2015), and to enhance exhibition of antitumor activities in prostate cancer (Ouyang et al., 2013).

Despite the proven antitumor activities of curcumin and piperine, the low solubility and the poor chemical stability of the compounds in water, largely limit their clinical applications. Targeted and triggered drug delivery systems accompanied by nanoparticle technology have been investigated as a prominent strategy to address these limitations (Bisht and Maitra, 2009; Ucisik et al., 2013b; Yallapu et al., 2013; Purpura et al., 2018; Wong et al., 2019). Polymeric nanoparticles (Anand et al., 2010; Bisht et al., 2010; Mahmood et al., 2015; Pachauri et al., 2015), cyclodextrin nanoparticles (Ndong Ntoutoume et al., 2016; Dash and Konkimalla, 2017b), liposomes (Li et al., 2005; Dutta and Bhattacharjee, 2017), mixed and copolymeric micelles (Gou et al., 2011; Wang et al., 2012; Li et al., 2015; Hevus et al., 2017), and solid lipid nanoparticles (Ucisik et al., 2013b; Jourghanian et al., 2016) are among the mostly applied curcumin and piperine nanoformulations (Naksuriya et al., 2014; Mahran et al., 2017). As a lipid-based, safe and surfactant-free system, emulsomes emerge as a promising drug delivery system among the alternatives.

Emulsomes are biocompatible vesicular systems comprising of a solid fat core surrounded by phospholipid multi-layers (Ucisik et al., 2013a). Owing a solid fat core, emulsomes can entrap higher amounts of lipophilic drug compounds and simultaneously offer a prolonged release time compared to emulsion formulations possessing a liquid, or fluidic, core. Previously, curcumin-loaded emulsomes, so-named CurcuEmulsomes, increased the solubility of the curcumin upto 10,000-fold and enabled delivery to the cells as the prolonged therapeutic effect of the formulation as verified on HepG2 cells *in vitro* (Ucisik et al., 2013b). Another recent study has also demonstrated that CurcuEmulsomes can be modified with certain surface moieties such as crystalline surface (S-) layer proteins to endow targeting feature to the nanocarrier (Ucisik et al., 2015a). These previous findings put emulsomes forward as prominent drug delivery system for poorly water-soluble therapeutic agents such as curcumin and piperine.

With the depicted approach, the present study formulates curcumin and piperine into emulsomes to enhance their limited bioavailability, and thus to achieve combinational anti-cancer effect on *in vitro* colon cancer model. The overall effect of combined therapy was studied through analysis on cell viability, cellular uptake, apoptotic cell death and cell cycle, as well as gene expression levels to further provide evidence how the two active molecules interact with HCT116 cancer cells in molecular basis.

## Materials and Methods

### Materials

Curcumin, piperine, glyceryl tripalmitate (tripalmitin, purity ≥ 99%), 1,2-dipalmitoyl-rac-glycero-3-phosphocholine (DPPC, ∼99%), Cholesterol (≥%99) were purchased from Sigma-Aldrich, Germany. Chloroform (≥99.8%) was obtained from Fluka Chemika, Germany. Dimethyl sulfoxide (DMSO) was purchased from Fisher BioReagents, United States. All chemicals were used as received without further purification.

3-(4,5-di-methyl-thiazol-2-yl)-5-(3-carboxy-methoxy- phenyl)-2-(4-sulfo-phenyl)-2H-tetrazolium (MTS)-assay (CellTiter96 AqueousOne Solution) was purchased from Promega, Southampton, United Kingdom. Annexin V-FITC Apoptosis Detection Kit was obtained from BD Pharmingen. Propidium iodide and RNase A were purchased from Sigma-Aldrich, Germany. Non-idet P-40 was obtained from AppliChem, Germany. 4,6-Diamidino-2-phenylindole dihydrochloride (DAPI) was purchased from Roche. pKH26 was obtained from Sigma-Aldrich, Germany.

### Synthesis of Curcumin- and Piperine-Loaded Emulsomes

**FIGURE 1:**
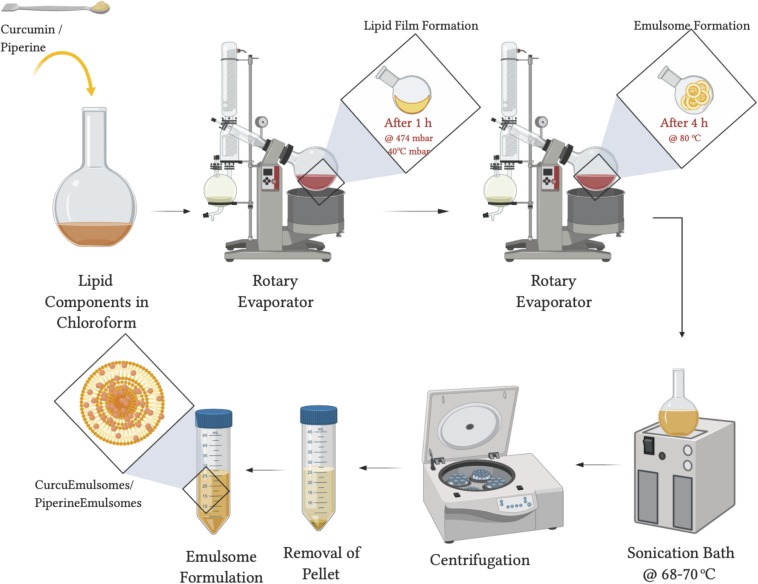
Graphical scheme for Curcu- and PiperineEmulsome preparation. This figure was created using BioRender (https://biorender.com/).

As illustrated in Figure 1, CurcuEmulsome and PiperineEmulsome formulations have been separately synthesized applying the procedure described before with slight modifications (Ucisik et al., 2013b, 2015a). Briefly, the rotary evaporation technique was used, where lipids including 20 mg tripalmitin, 2 mg dipalmitoyl phosphatidylcholine and 0.6 mg cholesterol together with curcumin (8 mg) or piperine (7 mg) were first dissolved in organic solvent, i.e., chloroform (2 mL). The solvent was completely removed, and dry lipid film was rehydrated with 5 mL aqueous solution. Ultrasonication bath at 70°C replaced the final extrusion step (Ucisik et al., 2013b, 2015a) to homogenize the particle size. To spin down unincorporated curcumin and piperine within the solution, preparations were centrifuged at 13,200 rpm (16,100 *g*) for 10 min. The CurcuEmulsome and Piperine-Emulsome suspension, i.e., the supernatant, was stored at 4°C until further characterization and cell culture studies. Empty emulsomes were prepared following the same procedure without adding curcumin and piperine.

### Characterization of Emulsome Formulations

#### Physicochemical Parameters: Size, Polydispersity, and Zeta-Potential

The mean particle size, size distribution [polydispersity index (PDI)] and zeta potential of the emulsome formulations were determined by the dynamic light scattering (DLS) method using a Zetasizer (Zetasizer Nano ZS, Malvern Instruments, Ltd., United Kingdom). Before measurement, the emulsome suspensions were diluted with 1 mM KCl solution to suitable concentrations. All analysts were performed in the auto-measuring mode at 25°C, and the results were obtained as the average value of triplicate samplings and measurements for each formulation.

#### Particle Size and Shape

A scanning electron microscope (Zeiss EVO-HD-15) was used for the size, shape, and morphology analysis of emulsome formulations. Before the imaging process, a short-term fixation procedure was applied as pre-treatment. Briefly, emulsome samples were placed onto an aluminum holder and left at 4°C overnight to dry. Dried samples were treated with PBS containing 2.5% glutaraldehyde for 15 min. PBS containing 2.5% glutaraldehyde was removed and samples washed with distilled water for three times in total along 10 min. Following gold sputtering (EM ACE200, Leica), the samples were observed under SEM (Zeiss EVO-HD-15).

#### Determination of Particle Concentration Using Nanoparticle Tracking Analysis (NTA)

Emulsome formulations were analyzed using a NanoSight NS300 instrument (Malvern Instruments) with a 488 nm laser. Samples were diluted to the suggested concentration range of the device, showing between 20 and 200 in frame. The NTA software is optimized to first identify and then track each particle on a frame-by-frame basis, and its Brownian movement is tracked and measured from frame to frame. The velocity of particle movement is used to calculate particle size by applying the two-dimensional Stokes–Einstein equation. The range of sizes that can be analyzed by NTA depends on the particle type—a low refractive index (e.g., emulsomes). Video capture was done at camera for 60 s intervals. At the end of each capture, sample was introduced to the flow cell to flush the previous portion of the sample. A total of three captures were taken for each sample. Samples were analyzed with appropriate threshold settings. Video capture and analysis was done using NTA software version 3.4.

#### Quantification of Curcumin and Piperine Encapsulated in Emulsomes

To quantify the curcumin content within the CurcuEmulsomes the absorbance measurement procedure was followed as described at Ucisik et al. (2013b). 1 mg/ml curcumin stock solution was prepared in DMSO. Standard solutions were prepared by successive dilution (0, 5, 10, 20, 50, 100 μg/ml) of the stock solution in a 96-well microplate (NEST Scientific, cat #701001). CurcuEmulsome samples were diluted with DMSO in 1:10 ratio. The absorbance of both CurcuEmulsome sample and the standards were measured at 430-nm wavelength using UV-vis spectrophotometer (Spectramax i3 Multi-Mode Microplate Reader Detection Platform). A standard curve was prepared from the values of standards. Curcumin concentration of CurcuEmulsomes was estimated by the read-out of the absorbance intensity and corresponding concentration on the standard curve.

Piperine content incorporated to the PiperineEmulsomes was estimated by a HPLC protocol described by Kozukue et al. (2007) with slight modifications (Kozukue et al., 2007). PiperineEmulsome samples were freeze-dried using lyophilization instrument (Labconco FreeZone 6 Freeze Dryer) and dissolved in ethanol. HPLC was carried out on a Waters 2695 Alliance 2998 PDA detector. The Waters Symmetry C18 column (75 mm^∗^4.6 mm i.d.) was packed with Inertsil ODS-3v (5 μm particle diameter). The column temperature was maintained constant at 25°C. Separation was achieved using a mixture of acetonitrile/0.5% formic acid in distilled water (30:70, v/v) as eluent with an isocratic flow rate with 1.0 mL/min at 25°C. A Shimadzu UV-vis detector (model SPD-10Avp) was set at 280 nm. The experiments were repeated as triplicates and the samples were protected from the light throughout the procedure.

#### Encapsulation Efficiency

Encapsulation efficiency of CurcuEmulsomes and PiperineEmulsomes were calculated according to the following equation:

$$EncapsulationEfficiency\left(\%\right)=\frac{W_{i\,n\,c\,o\,r\,p\,o\,r\,a\,t\,e\,d\,\,c\,o\,m\,p\,o\,u\,n\,d}}{W_{t\,o\,t\,a\,l}}\times 100\%$$

W_*incorporated compound*_: amount of curcumin/piperine in the emulsomes; W_*total*_: amount of curcumin/piperine used in formulation.

#### *In vitro* Drug Release

The procedure explained by Bisht et al. (2007) was applied to determine *in vitro* drug release profiles of curcumin and piperine from emulsomes (Bisht et al., 2007). Accordingly, 2 ml of CurcuEmulsome solution with 0.01M PBS (pH 7.4) was divided into 10 microcentrifuge tubes (200 μl in each tube). The tubes were kept in a thermo-shaker incubator (MTC-100, ThermoShaker Incubator, Hangzhou Miu Instruments, Co., Ltd.) that was set at 37°C for 0 min, 30 min, 1, 2, 3, 6, 12, 24, 48, and 72 h. At each time interval, one tube was removed and was centrifuged at 3000 *g* for 5 min (MicroCL 21R Microcentrifuge, ThermoScientific) to separate the released (i.e., free curcumin in the solution) from the loaded particles. The supernatant was collected and the pellet (released) curcumin re-dissolved in 300 μl DMSO and the absorbance was measured spectrophotometrically at 430 nm (Spectramax i3 Multi-Mode Microplate Reader Detection Platform, Molecular Devices).

The quantification of released piperine was measured by a HPLC system as described by Kozukue et al. (2007). After centrifugation, the pellet containing the released piperine was dissolved in ethanol and the tubes were stored at 4°C until all time intervals have been completed. HPLC was carried out on a Waters 2695 Alliance 2998 PDA detector as described previously in Section “Determination of Particle Concentration Using Nanoparticle Tracking Analysis (NTA).” The experiments were repeated as triplicates and the samples were protected from the light throughout the procedure.

### Cell Culture

HCT116 (CCL-247) (human colon carcinoma) cell line was purchased from American Type Culture Collection (ATCC) (Rockville, MD, United States). Cells were cultured in Dulbecco’s modified Eagle’s (DMEM) medium supplemented with 10% fetal bovine serum (FBS) (Invitrogen), 100 units/mL of penicillin, 100 μg/mL of streptomycin and amphotericin (Biological Industries, Beit HaEmek, Israel) and grown in plastic flasks (Rutherford, NJ, United States).

### Cell Viability

Following the treatments, cell viabilities of HCT116 cells were determined by MTS assay. First, HCT116 cells were seeded in 96-well plates with a density of 6,000 cells per well. After 24 h incubation, cells were treated with free curcumin (in DMSO), free piperine (in DMSO), CurcuEmulsome, PiperineEmulsome and their combinations in different concentrations. Following the incubation with CurcuEmulsome and PiperineEmulsome for 24, 48 and 72 h, cells were incubated in 10% MTS containing DMEM solution. Absorbance at 490 nm was detected using a microplate spectrophotometer (xMark-Biorad, Canada). Data were analyzed with GraphPad Prism 6.01 software and IC_50_ values were determined.

### Cell Morphology and Cell Uptake Analysis Using Confocal Laser Scanning Microscopy Analysis

HCT116 (3 × 10^5^ cells/well) cells were seeded on 6 well plate (SPL Lifesciences, South Korea). After 24 h incubation, cells were treated with 25 μM CurcuEmulsome. After 2 and 24 h incubations, cells were washed twice with 1 mL of PBS, and incubated with pKH-26 red dye according to manufacturer’s protocols. Then, samples were fixed with freshly prepared 4% paraformaldehyde for 10 min, then washed three times with PBS and stained with 5 μg/ml DAPI. Each samples was visualized with a Zeiss LSM 800 confocal microscope using FITC filter (excitation at 488 nm; emission at 525 nm) and Alexa Fluor 555 filter (excitation at 553 nm; emission at 568 nm) with a 40x objective. Using BD FACSCalibur, fluorescence intensity at FL1-H (excitation at 488 nm; emission at 525 nm) for cells incubated with CurcuEmulsome for 2, 4, 6, 24, 48, and 72 h were measured. Three-dimensional (3D) reconstructions created from X, Y, and Z sections of complete Z-stack images of confocal microscope. An upright Zeiss Axiovert A1 microscope was used to visualize the cell morphology of HCT116 cells.

The effect of PiperineEmulsomes on CurcuEmulsome’s uptake was studied separately with an independent setup. Accordingly, HCT116 (2 × 10^4^ cells/well) cells were seeded on 8 well chamber slides (SPL Lifesciences, South Korea) for 72 h. After 24 h incubation, cells were treated with 25 μM CurcuEmulsome, 7 μM PiperineEmulsome and their combinations. Then, cells were washed twice with 300 μl of PBS, fixed with freshly prepared 4% paraformaldehyde for 10 min, then washed three times with PBS and stained with 5 μg/ml of DAPI. Each sample was visualized with a Zeiss LSM 780 confocal microscope using FITC filter (excitation at 488 nm; emission at 525 nm) with a 40x objective.

### Apoptosis

The apoptosis response of HCT116 cells to CurcuEmulsome and PiperineEmulsome combination therapy was analyzed by Annexin V kit in flow cytometer. Briefly, 300,000 cells/well were seeded on 6 well plates and after 24 h incubation media were aspirated and treated with 25 μM CurcuEmulsome and 7 μM PiperineEmulsome and their combinations. After 48 and 72 h, cells were harvested and 1 × 10^5^ of each sample were treated with Annexin V and propidium iodide (PI) for 15 min according to manufacturer’s protocol. Samples were analyzed then immediately by FACSCalibur (BD Biosciences). Data from at least 20,000 cell counts were collected for each data file. Gating was set properly to exclude cell debris, cell doublets, and cell clumps.

### Cell Cycle Analysis

Cell cycle distribution of HCT116 cell line was detected with help of flow cytometer for 24, 48, and 72 h to obtain comparable results. 300,000 cells per well were seeded in 6-well plates. Treatments were done after 24 h to allow the attachment of the cells to the plates. After 24, 48 and 72 h, cells were fixed by 70% ethanol solution, washed twice with PBS and incubated for 30 min with 0.01% non-idet P-40, 10 μg/mL RNase A, and 8 μg/ml Propidium iodide mixture. 20,000 cells were analyzed by Guava easyCyte Flow Cytometer (Merck Millipore, Germany) and the percentages of the cell populations in G0/G1, S and G2/M phases were determined.

### Quantitative PCR

Primers for Caspase 3 F: 5′-GAGGCGGTTGTAGAAGAGTT CGTG-3′ and Caspase 3 R: 5′-TGGGGGAAGAGGCAG GTGCA-3′ were designed by using Primer-BLAST online software from the National Center for Biotechnology (Bethesda, MD, United States) and synthesized by Macrogen (Seoul, South Korea). Total RNAs from all treated and non-treated samples were isolated using TRIzol reagent (Thermo Fisher Scientific, United States) according to the manufacturer’s instructions. High Fidelity cDNA-synthesis kit (Roche, United States) was used to synthsize the cDNA’s. QuantiTect SYBR Green PCR kit (Qiagen, United States) was used for the quantitative polymerase chain reaction (qPCR) to quantify mRNA levels of the genes. The housekeeping gene, 18sRNA (Hs_RRN18S_1_SG QuantiTect Primer Assay) was used for normalization of data. All RT-PCR experiments were done using iCycler RT-PCR system (Bio-Rad, Hercules, CA, United States).

### Statistical Analysis

GraphPad Prism Software (version 6.01) was used to perform statistical analysis. Error bars represent standard error of the mean and the data sets were compared using two-way ANOVA and Student’s *t*-test. Differences were considered statistically at (^∗^) *P* ≤ 0.05, (^∗∗^) *P* ≤ 0.01, (^∗∗∗^) *P* ≤ 0.001, (^****^) *P* ≤ 0.0001.

## Results

### Characterization

#### Size and Zeta Potential of CE and PE

**FIGURE 2:**
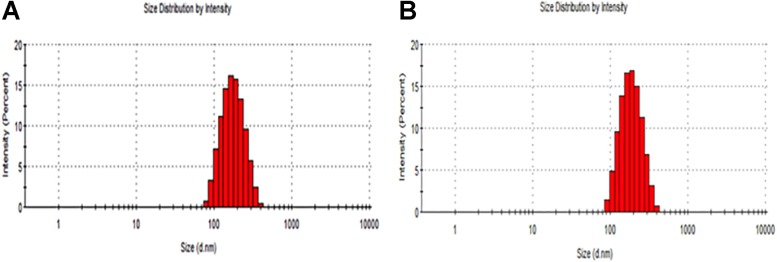
Particle size distribution of **(A)** CurcuEmulsome and **(B)** PiperineEmulsome formulations.

The particle size distribution (DLS) and zeta potential characteristics (Phase Analysis Light Scattering; M3–PALS) of formulations were determined for more than five distinct CurcuEmulsome and PiperineEmulsome formulations using Zetasizer Nano ZS (Malvern Instruments, Ltd., United Kingdom). As depicted in Table 1, the average diameter of CurcuEmulsomes was determined as 184.21 ± 13.30 nm (polydispersity index of 0.19; conductivity of 0.0178 ± 0.003 mS/cm), while PiperineEmulsomes were found to have a larger average size: 248.76 ± 50.8 nm (polydispersity index of 0.250; conductivity of 0.019 ± 0.002 mS/cm) – where the plus-minus signs indicate the margin of average sizes of numerous CurcuEmulsome and PiperineEmulsome formulations made of the same composition. The particle size distribution curves indicated that the size of the emulsome formulations largely vary between 100 and 300 nm (Figure 2). Analogous to PiperineEmulsomes, the mean diameter of empty emulsomes was determined as 239.12 ± 51.69 nm. All emulsome formulations displayed a negative zeta potential; however, while empty emulsomes (−16.74 ± 4.56 mV) and PiperineEmulsome (−20.46 ± 6.85 mV) shared comparable values, CurcuEmulsomes distinguished with a higher negative charge (−34.23 ± 4.34 mV), which is further attributed to the contribution of molecular negative charge of curcumin to the formulation. Owing a more negative zeta potential, CurcuEmulsomes seem to distinguish from empty emulsomes and PiperineEmulsome with a smaller average particle size. The polydispersity index data shows a linearity between negative zeta potential and monodispersity of the formulations (Table 1).

**TABLE 1:** Average values for particle size, polydispersity index (PDI) and zeta potential.

**Formulations**	**Particle diameter**	**Polydispersity**	**Zeta potential**
	**(nm)**	**index**	**(mV)**
Emulsome^a^	239.12 ± 51.69	0.335 ± 0.09	−16.74 ± 4.56
CurcuEmulsome^b^	184.21 ± 13.30	0.197 ± 0.06	−34.23 ± 4.34
PiperineEmulsome^c^	248.76 ± 50.8	0.250 ± 0.12	−20.46 ± 6.85

*^*a*^More than five separate samples with average conductivity of 0.170 ± 0.007 mS cm^–1^. ^*b*^More than five separate samples with average conductivity of 0.0178 ± 0.003 mS cm^–1^. ^*c*^More than five separate samples with average conductivity of 0.019 ± 0.002 mS cm^–1^. Standard deviations given by the ± values correspond to the average standard deviation of all measurements, where *n* ≥ 3.*

#### Microscopy Analysis for Shape and Particular Dispersity

**FIGURE 3:**
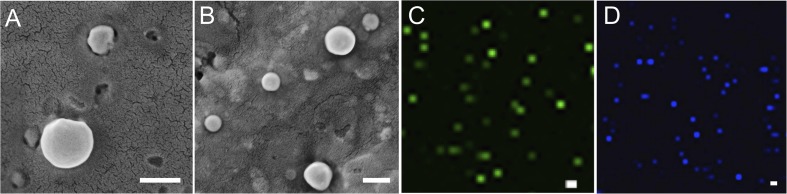
SEM images of **(A)** CurcuEmulsomes, and **(B)** PiperineEmulsomes. Confocal image of **(C)** CurcuEmulsomes and **(D)** PiperineEmulsomes. Scale bars correspond to 200 nm.

SEM analysis demonstrated that the spherical character of the emulsome particles had conserved after encapsulation of curcumin as well as piperine (Figures 3A,B). Each particle in the formulation appears to have the same rough, spherical surface morphology indicating a homology in their characteristic structure where the outermost surface is stabilized smoothly by phospholipid bilayers. Confirming the DLS analysis, SEM images verified that the sizes of both CurcuEmulsome and PiperineEmulsomes vary between 100 and 300 nm.

Taking advantage of the autofluorescence properties of Curcumin and Piperine, the formulations were also analyzed by laser scanning confocal microscopy (Zeiss LSM780, Turkey). Unlike the SEM analysis, where the dried samples were analyzed, the confocal analysis enabled the evaluation of the behavior of the formulation in aqueous environment. Both CurcuEmulsome and PiperineEmulsome showed dispersed characters in aqueous solution (i.e., water). Despite not being as precise as SEM, confocal analysis likewise suggested the average size of samples to be around 200 nm (Figures 3C,D).

#### Quantification of Emulsome Formulations

The amount of CurcuEmulsomes and PiperineEmulsomes were described in terms of curcumin and piperine concentrations, respectively. As previously described at Ucisik et al. (2013b) CurcuEmulsomes achieve curcumin encapsulation up to 0.10 mg/ml. CurcuEmulsomes synthesized in this study showed curcumin concentrations in average 0.07 ± 0.02 mg/ml. While the maximum piperine content of PiperineEmulsome formulation was achieved as 0.09 mg/ml – which is very similar to curcumin encapsulation – the average piperine concentration of the formulation remained around 0.05 ± 0.02 mg/ml along the study.

CurcuEmulsome and PiperineEmulsomes concentrations were alternatively defined as particles per mL with the help of NTA system. According to the NTA data (Supplementary Figure 1), 1 μM curcumin concentration of CurcuEmulsomes corresponds to approximately 6.7 × 10^8^ particles per mL (1:150 dilution from the stock CurcuEmulsome); and 1 μM piperine concentration of PiperineEmulsome corresponds to 4.5 × 10^8^ particles per mL (1:220 dilution from the stock PiperineEmulsome). Cells in emulsome control group was treated with 1.7 × 10^9^ particles per mL that corresponds to particle concentration of 25 μM CurcuEmulsomes.

#### Encapsulation Efficiency of CurcuEmulsomes and PiperineEmulsomes

In synthesis of formulations, curcumin and piperine were used in excess compared to other lipid components to enable maximum amount of encapsulation of the lipophilic compounds. Encapsulation efficiencies of curcumin and piperine inside emulsomes were calculated as 4.4 and 3.6% for CurcuEmulsomes and PiperineEmulsomes, respectively.

#### Drug Release Profiles

**FIGURE 4:**
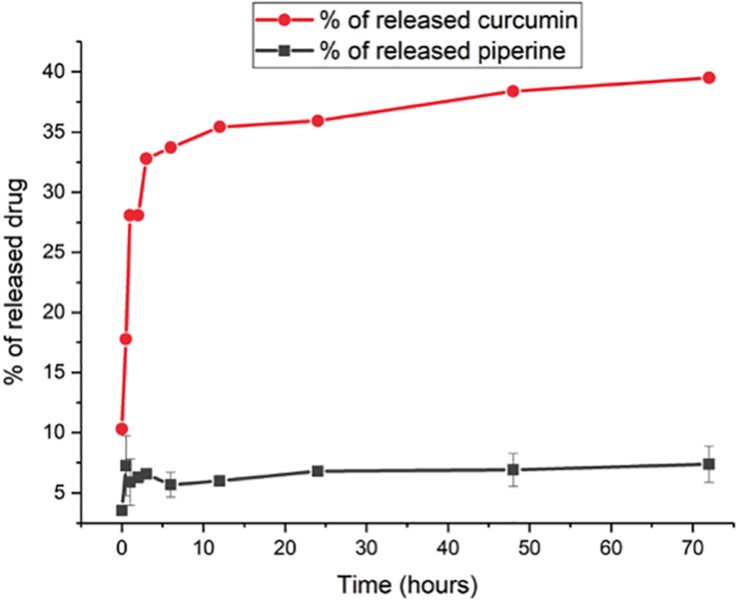
*In vitro* drug release profiles of CurcuEmulsomes (red; 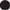) and PiperineEmulsomes (gray; 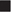). Values reported are mean values plus/minus standard deviation with number of measurements equal to 3.

**FIGURE 5:**
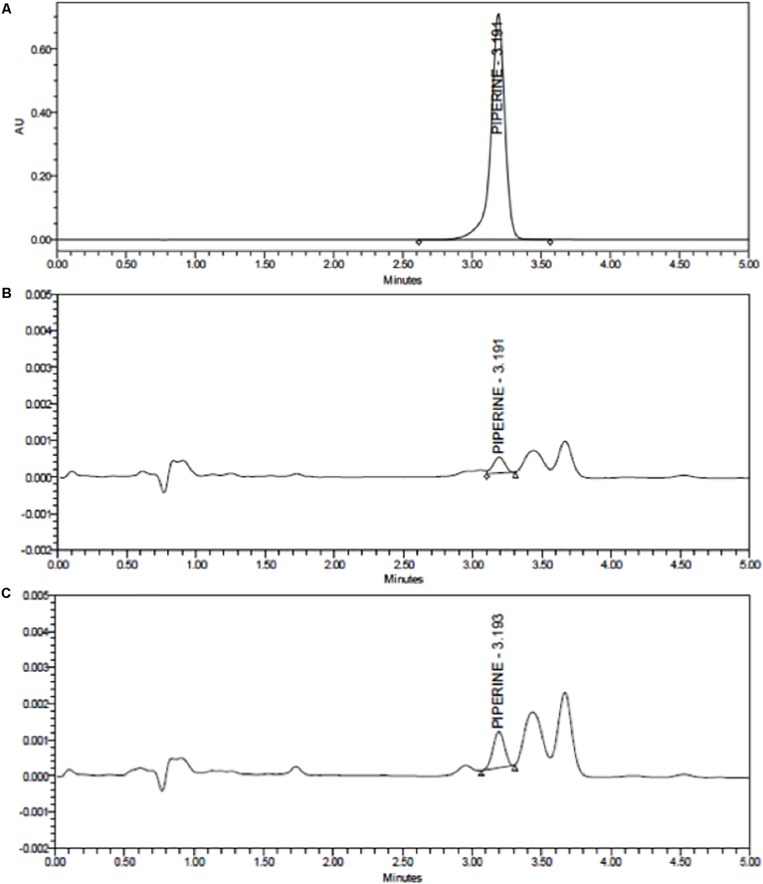
HPLC chromatograms of **(A)** standard piperine solution, **(B)** released total piperine in 1 h, and **(C)** released total piperine in 72 h.

The release profiles of both formulations are presented in Figure 4. Two-way ANOVA was applied to analyze the data statistically. Accordingly, curcumin release is found significant (*P* ≤ 0.0001) for all time points, whereas piperine release is significant only for the following time points: 3 h (*P* ≤ 0.05), 24 h (*P* ≤ 0.05), 48 h (*P* ≤ 0.01), and 72 h (*P* ≤ 0.01). CurcuEmulsomes released nearly 25% of its curcumin content within the first 3 h, which after the release slows down and reaches to the levels around 32, 35, and 40% after 24, 48, and 72 h, respectively. On the other hand, the release of piperine from the PiperineEmulsomes occurs in the first 6 h as around 7% and remains almost constant around 7 and 7.5% along 72 h. The HPLC chromatogram displaying piperine amount released from PiperineEmulsomes after 1 h (Figure 5B) and 72 h (Figure 5C), notified the presence of further compounds that were not present in the standard piperine solution (Figure 5A). The presence of additional peaks is attributed to the isomerization of piperine and will be addressed further in Section “Discussion.”

### Effect of CurcuEmulsome and PiperineEmulsome on Cell Viability

HCT116 cells were treated with free curcumin, free piperine, CurcuEmulsome, and PiperineEmulsome at various concentrations for 72 h. Curcumin concentrations in both free and CurcuEmulsome form were set in range of 2.5 and 50 μM; whereas piperine concentrations in both free and PiperineEmulsome form were in range of 0.7 and 14 μM. Curcumin and piperine were also applied alone as well as combined to HCT116 cells within prementioned concentration ranges. The cells were analyzed at 24, 48, and 72 h using MTS cell viability assay. DMSO contents within the total cell media containing free curcumin and free piperine samples were kept below 0.15% v/v throughout the experiment to avoid any influence of DMSO to cell viability. To keep track on the influence of the presence of the inorganic solvent (i.e., DMSO), one control group was treated with 0.15% v/v DMSO (data not shown).

**FIGURE 6:**
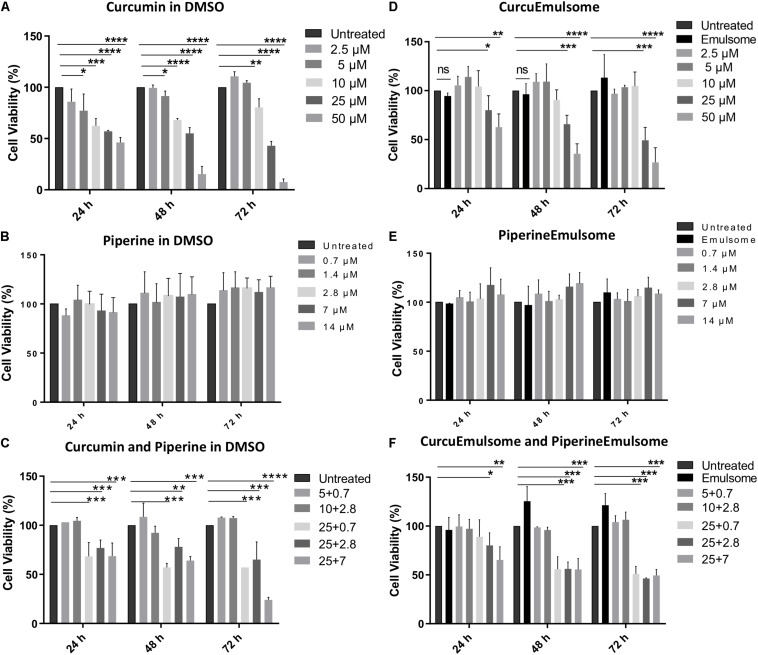
MTS cell proliferation analysis of colon cancer cell line HCT116 treated with **(A)** free curcumine (in DMSO), **(B)** free piperine (in DMSO), **(C)** curcumin and piperine combinations, **(D)** CurcuEmulsome, **(E)** PiperineEmulsome, and **(F)** their combinations. Cytotoxity of the drug delivery system alone to HCT116 cells were investigated at various concentrations for 24, 48, and 72 h compared to untreated cells. Data represents mean (*n* = 3) ± SD (*P ≤ 0.05, **P ≤ 0.01, ***P ≤ 0.001, ****P ≤ 0.0001).

As shown on cell viability data (Figure 6), all treatment groups including (i) free curcumin, (ii) CurcuEmulsome, (iii) combinations of free piperine and free curcumin and (iv) combination of CurcuEmulsome and PiperineEmulsome reduce cell viability in dose and time dependent manner. Free curcumin reduced cell viability significantly at concentrations 10, 25, and 50 μM (Figure 6A), whereas free piperine displayed no significant effect on viability of HCT116 cells at any concentration (Figure 6B). When cells were treated combined with both 25 μM free curcumin and 7 μM free piperine, cell viability became as low as 20% after 72 h (Figure 6C). Similar to free curcumin, CurcuEmulsome treatment caused a gradual decrease in viability of HCT116 cells as the concentration of the CurcuEmulsomes in the growth media was increased to 10, 25, and 50 μM curcumin concentrations (Figure 6D). In contrary to CurcuEmulsomes, yet parallel to free piperine, PiperineEmulsomes produced no significant change in viability of HCT116 cells (Figure 6E). The obtained results indicated that combination of 7 μM PiperineEmulsome with 25 μM CurcuEmulsome has the highest potential to induce cancer cell death (Figure 6F).

**FIGURE 7:**
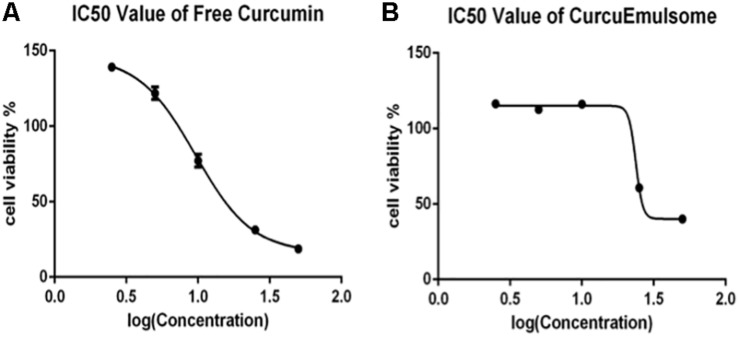
Cell viability versus logarithmic concentration data for estimation of IC50 values: **(A)** free curcumin and **(B)** CurcuEmulsome against HCT116 cells for 72 h.

Using these data on Figure 6, IC50 values of free curcumin and CurcuEmulsomes were estimated against HCT116 cells as 11.08 ± 1.31 and 19.69 ± 3.27 μM for 72 h, respectively (Figure 7).

### Uptake of CurcuEmulsome and PiperineEmulsome by HCT116 Cells

**FIGURE 8:**
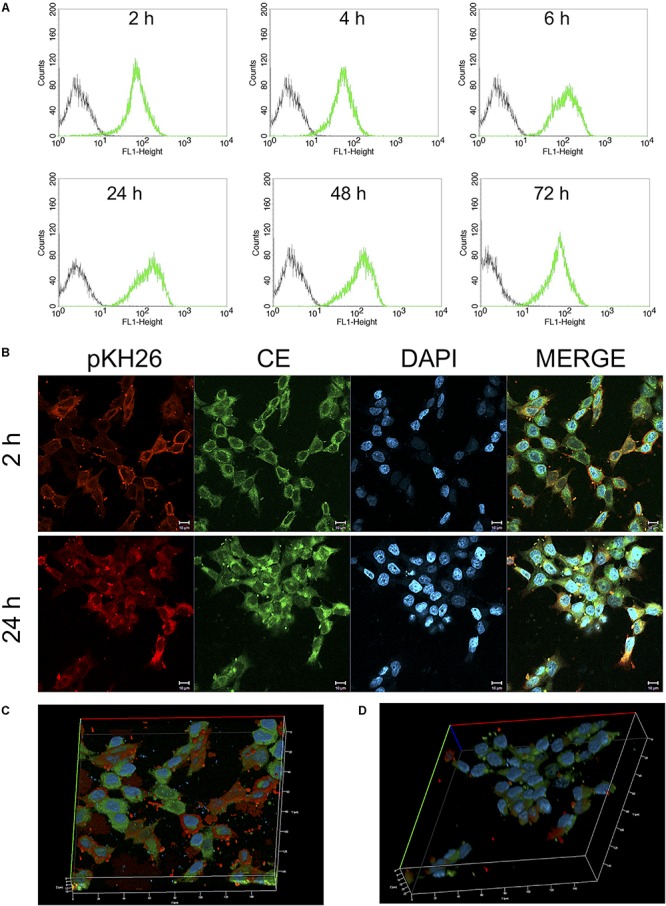
**(A)** Cellular uptake of 25 μM CurcuEmulsome by HCT116 cells by flow cytometry. Cells were incubated for 2, 4, 6, 24, 48, and 72 h at 37°C followed by measurement of fluorescence at FL-1 (excitation/emission, 488:525 nm). **(B)** Confocal microscopy images after incubation of 25 μM CurcuEmulsome with HCT116 cells at 37°C for 2 and 24 h. Images were taken using 40x objective and scale bars correspond to 10 μm. **(C,D)** Z-stack three-dimensional image reconstruction of HCT116 cells treated with CurcuEmulsomes for 2 and 24 h, respectively.

Both flow cytometry and confocal microscopy were conducted to monitor the uptake of CurcuEmulsomes into HCT116 cells. Flow cytometry results demonstrated that the cellular binding of CurcuEmulsomes by HCT116 cells were 69.7 ± 13.6, 73.5 ± 6.3, 79.3 ± 11.8, 95.9 ± 0.1, 97.3 ± 0.5, and 98.0 ± 0.1% for 2, 4, 6, 24, 48, and 72 h, respectively (Figure 8A). In parallel, confocal microscopy results confirmed the uptake of the formulation. The autofluorescence properties of curcumin (Ucisik et al., 2013b) allowed tracking the internalization of emulsomes by confocal microscopy. Cell nuclei were labeled with DAPI to further analyze the localization of the formulation within the cell. Confocal microscopy results showed that HCT116 cells treated with CurcuEmulsomes at 24 h had the highest fluorescence intensity compared to 2 h incubation (Figure 8B). Z-stack three-dimensional (3D) image reconstruction further provided further information on internal localization of CurcuEmulsomes. Total 30 z-stacks were used to assemble the 3D images (Figures 8C,D).

**FIGURE 9:**
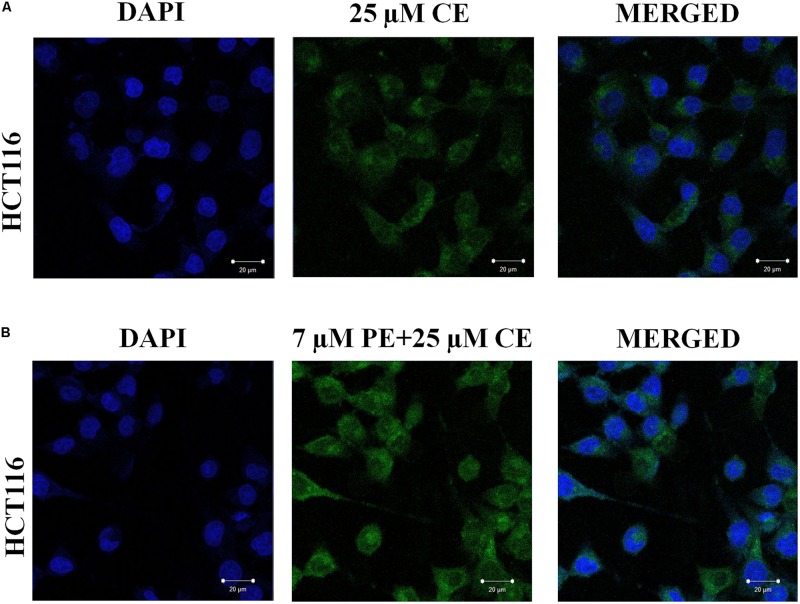
Confocal microscopy images showing the uptake of CurcuEmulsomes by HCT116 cells in the absence and in the presence of PiperineEmulsomes. Cells were treated with **(A)** 25 μM CurcuEmulsome or **(B)** 25 μM CurcuEmulsome plus 7 μM PiperineEmulsome for 24 h. Image was taken under 40x objective. Bars correspond to 20 μm.

Effect of PiperineEmulsomes on CurcuEmulsome’s uptake was studied in a dual therapy approach where HCT116 cells were treated with both CurcuEmulsomes and PiperineEmulsomes *in vitro*. Cellular uptake of CurcuEmulsome in the absence and in the presence of PiperineEmulsomes was monitored by confocal microscopy, as the autofluorescence properties of curcumin (Ucisik et al., 2013b) and piperine (Patel and Vyas, 2012) allowed the tracking. Internalization of CurcuEmulsomes (25 μM) was observed in both absence and presence of PiperineEmulsomes (7 μM) (Figure 9). Higher fluorescence intensity of CurcuEmulsomes in combinational treatment was attributed to occurrence of a higher uptake in the presence of PiperineEmulsomes.

### Effects of CurcuEmulsomes and PiperineEmulsomes on Morphology of HCT116 Cells

**FIGURE 10:**
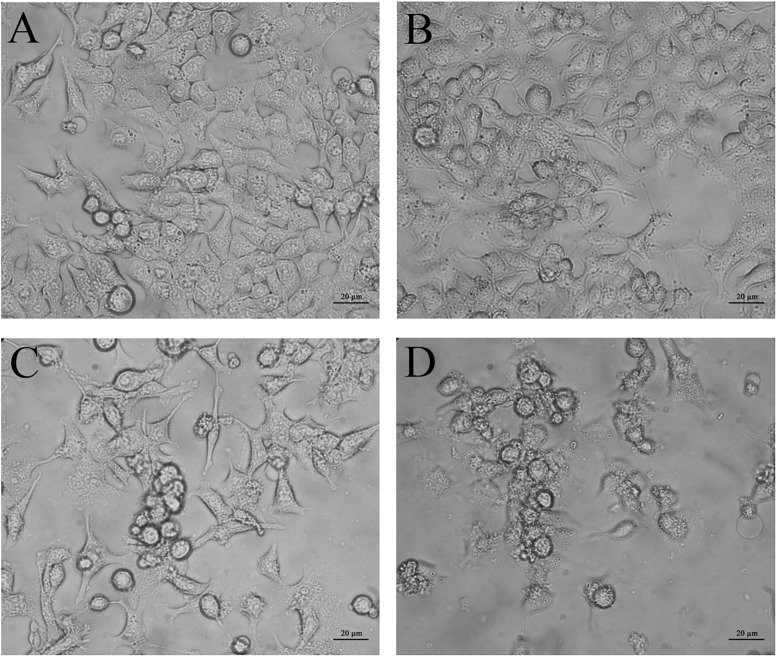
Microscopy images of **(A)** negative control, **(B)** PiperineEmulsome, **(C)** CurcuEmulsome, and **(D)** PiperineEmulsome plus CurcuEmulsome treatment groups after 48 h. HCT116 cells at negative control and PiperineEmulsome groups preserved their morphology throughout the study whereas cells treated with CurcuEmulsome or CurcuEmulsome plus PiperineEmulsomes showed round shape after 48 h exposure. Bars correspond to 20 μm.

Phase contrast inverted microscopy was used to visualize morphological changes of HCT116 cells mediated by CurcuEmulsome and PiperineEmulsome treatment. Untreated cells displayed flattened attachment on the surface (Figure 10A), which did not alter when cells were treated with 7 μM PiperineEmulsome (Figure 10B). 25 μM CurcuEmulsome treatment (Figure 10C) as well as its combinations 7 μM PiperineEmulsome (Figure 10D), on the other hand, changed the surface morphology and attachment of cells dramatically. The flat surface morphology has transformed to round spherical character and cells departed from each other as a consequence of this transformation in morphology.

### Effects of CurcuEmulsome and PiperineEmulsomes on Cell Cycle

**FIGURE 11:**
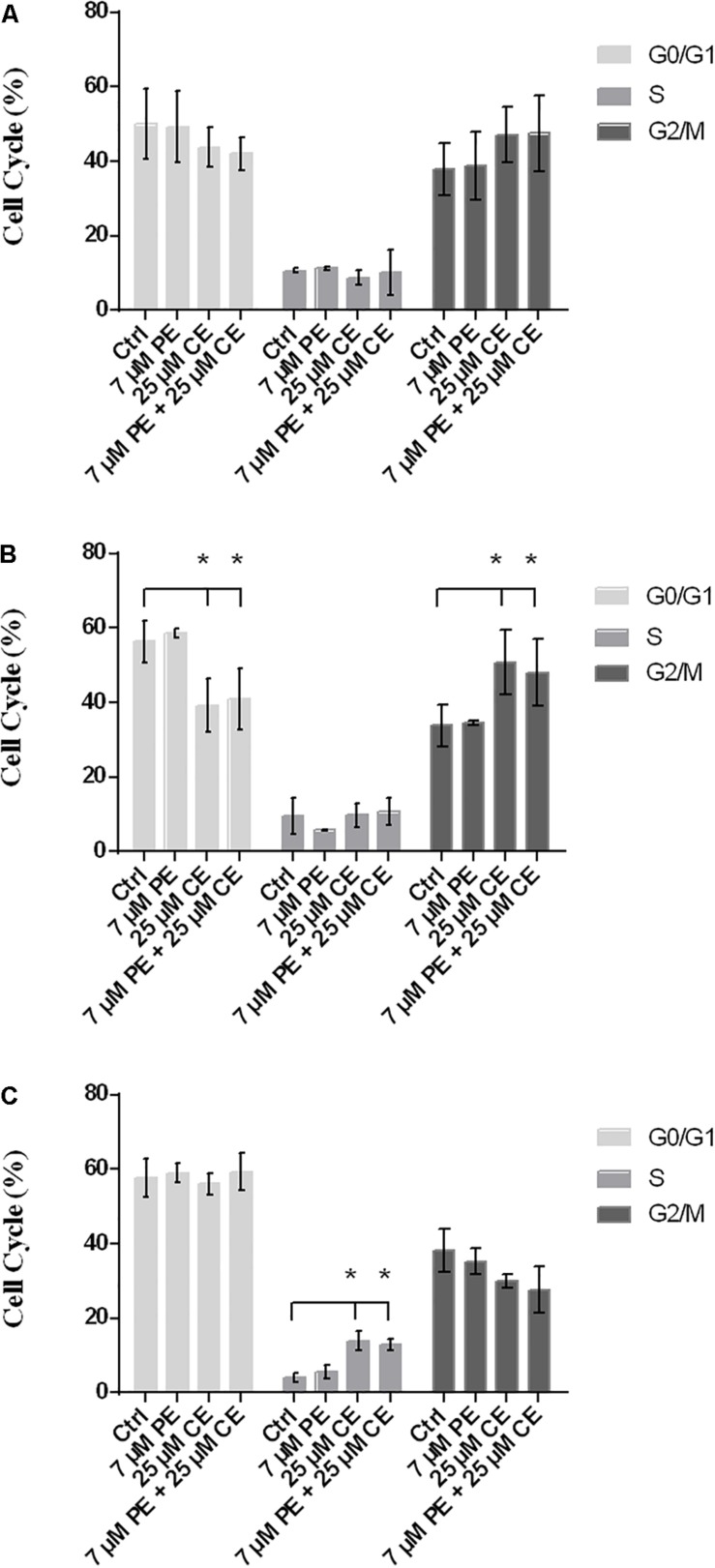
Cell cycle progression in HCT116 cells upon treatment with CurcuEmulsomes and PiperineEmulsomes. The HCT116 cells were treated with 25 μM CurcuEmulsome, 7 μM PiperineEmulsome and their combinations for **(A)** 24 h, **(B)** 48 h, and **(C)** 72h. *n* = 3 independent experiments were carried out. *P ≤ 0.05 indicates significant difference from respective control.

Flow cytometry with PI staining was performed to inspect the effect of curcumin on cell cycle progression in HCT116 cells. The proportion of cell population in G2/M phase was found to vary dependent on the treatment, indicating a cell cycle arrest induced in G2/M phase. While the frequency of G2/M cell cycle phase was for the control group 37.9 ± 6.8% after 24 h, it was found as (i) 38.7 ± 9.1% for 7 μM PiperineEmulsome treatment group, (ii) 47.1 ± 7.4% for 25 μM CurcuEmulsome treatment group, and (iii) 47.4 ± 10.2% when 7 μM PiperineEmulsome was co-delivered with 25 μM CurcuEmulsome. Parallel to the findings in the cell viability studies, these results suggest that (i) PiperineEmulsome alone does not alter the cell behavior in terms of cell cycle frequencies; (ii) consistent with previous studies (Toden et al., 2015) the delivery of curcumin – in CurcuEmulsome formulation – induce G2/M arrest in HCT116 cell line; (iii) Co-Delivery of PiperineEmulsome with CurcuEmulsome reduces the effect of CurcuEmulsome in terms of G2/M-phase cell cycle arrest, while leading to increase S phase cell populations (from 8.7 ± 2.0 to 10.2 ± 6.1%) (Figure 11A). The HCT116 cell population in G0/G1 phase was decreased both when treated with CurcuEmulsome alone (from 50.0 ± 9.5 to 43.9 ± 5.4%) and in combination with PiperineEmulsome (from 50.0 ± 9.5 to 41.9 ± 4.5%) as compared with that in the control cells. When the cell cycle frequencies at 48 h of treatment was analyzed, it can be seen that the cell population in G2/M phase is (i) 33.8 ± 5.5% for control group; (ii) 34.6 ± 0.4% for 7 μM PiperineEmulsome treatment group; (iii) 50.8 ± 8.7% for 25 μM CurcuEmulsome treatment group; and (iv) 48.0 ± 8.9% for cells treated combined with 7 μM PiperineEmulsome and 25 μM CurcuEmulsome (Figure 11B). At 72 h, the cell population in G2/M phase became (i) 38.3 ± 5.7% for control group; (ii) 35.3 ± 3.5% for 7 μM PiperineEmulsome treatment group; (iii) 29.9 ± 1.8% for 25 μM CurcuEmulsome treatment group; and (iv) 27.7 ± 6.1% for combined treatment of PiperineEmulsome and CurcuEmulsome (Figure 11C). Overall, these results indicate a decrease in the cell population at G2/M phase (i) from 47.1 ± 7.3 to 29.9 ± 1.8% for CurcuEmulsome treatment, and (ii) from 47.4 ± 10.2 to 27.7 ± 6.1% for the combined treatment.

### Effects of CurcuEmulsome and PiperineEmulsome on Apoptotic Cell Death

**FIGURE 12:**
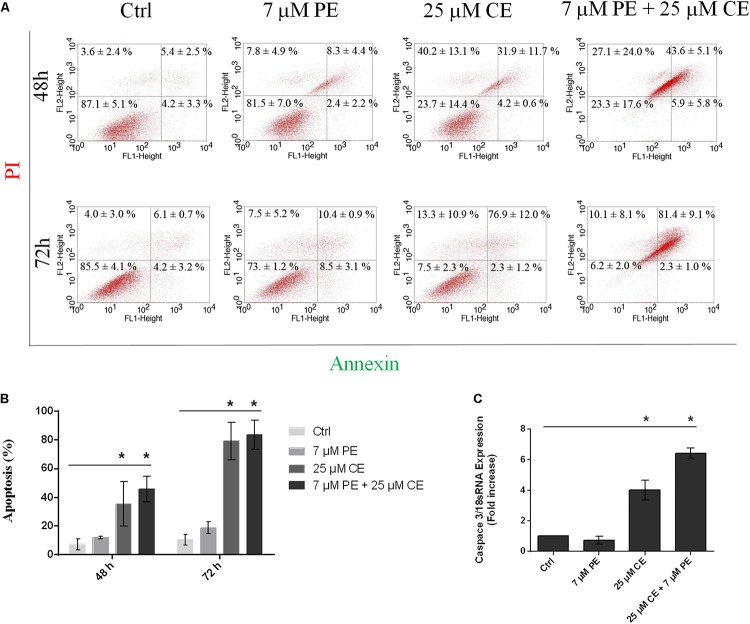
Apoptotic cell death of HCT116 cells upon treatment with 25 μM CurcuEmulsome, 7 μM PiperineEmulsome and their combinations for 48 and 72 h. **(A)** Representative histogram data of Annexin V-FITC/PI flow cytometer analysis, and **(B)** apoptotic cell percentage were presented. **(C)** Fold increase of relative caspase3/mRNA expression using real time PCR in HCT116 cells treated with 25 μM CurcuEmulsome, 7 μM PiperineEmulsome and their combinations. The data represents three independent experiments and *P* ≤ 0.05 (*) shows significant difference from respective control.

The apoptotic cell deaths upon treatment of HCT116 cells with CurcuEmulsome, PiperineEmulsome and their combinations were analyzed by Annexin V flow-cytometric assay. HCT116 cells were treated with (i) 25 μM CurcuEmulsome, (ii) 7 μM PiperineEmulsome and (iii) their combinations for 48 and 72 h, and apoptotic cell percentage were examined by counting the cells at early apoptosis (i.e., Annexin-V: positive, PI: negative) and late apoptosis (Annexin-V: positive, PI: positive) stages (Figure 12A). As shown in Figure 12B, population fraction of apoptotic cells was found to be around (i) 7.2 ± 3.9% for control groups; (ii) 10.7 ± 2.3% for PiperineEmulsome treatment group; (iii) 36.1 ± 14.1% for CurcuEmulsome treatment group; (iv) 49.6 ± 9.1% for combined treatment group, at 48 h; and around (i) 10.4 ± 3.8% for control groups; (ii) 18.9 ± 4.0% for PiperineEmulsome treatment group; (iii) 79.2 ± 13.2% for CurcuEmulsome treatment group; (iv) 83.7 ± 10.0% for combined treatment group, at 72 h.

### Effect of CurcuEmulsome and PiperineEmulsome on Caspase3 Gene Expression

Upon treatment of HCT116 cells with 25 μM CurcuEmulsome, 7 μM PiperineEmulsome and their combinations, the relative caspase3/18sRNA mRNA expressions of cells were analyzed using real-time PCR for 72 h. The results showed that, HCT116 cells treated with curcumin and combination overexpressed caspase3 gene four and six times, respectively (Figure 12C). Observed caspase3 overexpression is consistent with Annexin V/PI flow cytometry data (Figures 12A,B) indicating incidence of higher cell death when CurcuEmulsome and PiperineEmulsome are together applied.

## Discussion

Curcumin is a natural polyphenol with characteristic anti-cancer properties. The low solubility and the poor chemical stability of the compound prevent curcumin in its free form to reach effective blood serum concentrations and, as a consequence, necessitates the use of nanomedical approaches enabling its delivery to the cancer cells. As previously reported, emulsomes with their solid lipid inner matrix offer certain advantages for low water–soluble drug molecules such as curcumin (Ucisik et al., 2013b, 2015a). High load capacity, improved stability, prolonged release profile and biological safety. Cell uptake and accessibility for further surface modifications are the most prominent advantages that bring emulsomes among the alternative drug delivery systems one step forward (Ucisik et al., 2013a, 2015a,b). Making use of these promises, the current study applies emulsomes to carry both curcumin and piperine, i.e., two lipophilic natural polyphenols compounds displaying improved anti-cancer properties. The synergistic behavior of piperine together with curcumin for certain cancer types is known (Kakarala et al., 2010; Patial et al., 2015); however, the use of emulsomes for their delivery to the cancer cells was previously not reported. This study is featured by being the first showing the effects of curcumin and piperine on HCT116 colon cancer cells when delivered together within emulsomes.

### Characterization Studies of CurcuEmulsomes and PiperineEmulsomes

#### Physicochemical Properties

The results suggest that composed of tripalmitin surrounded by phospholipids emulsome successfully entraps both curcumin and piperine inside its inner solid matrix. Establishing a stable dispersion, CurcuEmulsomes and PiperineEmulsomes are spherical in shape and possess a smooth phospholipid outer surface (Figure 3). DLS analysis determined the average diameters of CurcuEmulsomes and PiperineEmulsomes as 184.21 ± 13.30 and 248.76 ± 50.8 nm, respectively (Table 1), where the plus-minus signs indicate the margin of average diameters, not the range of particle size, which vary between 100 and 300 nm (Figure 2). For drug delivery approaches, the size of nanocarriers is crucial parameter for the rates and mechanism of the cell uptake (Lee et al., 2010; Oh et al., 2011). It has been suggested that particles smaller than 1 μm can undergo capillary distribution and uniform perfusion at the desired target site (Arias et al., 2001). Nanocarriers much less than 400 nm are able to cross vascular endothelia and accumulate at the tumor site via the EPR effect (Gullotti and Yeo, 2009; Steichen et al., 2013). In addition He et al. (2010) have suggested that NPs with slight negative charges and particle size of 150 nm have a higher tendency to accumulate in tumor more efficiently *in vivo* (He et al., 2010). Therefore, the size distribution in range between 100 and 300 nm were evaluated to be suitable for our emulsome formulations.

Zeta potential also influences the cellular uptake and the efficacy of therapy. In general, it has been accepted that cationic particles show better internalization rates than their negative or neutral counterparts due to the negatively charged behavior of the cell membrane (Zolnik et al., 2010; Albanese and Chan, 2011; Cai et al., 2011). However, positively-charged cationic nanoparticles possess higher toxicity concerns than their neutral- or negatively-charged systems as a toxicity was observed with cationic emulsomes on HepG2 cancer cell line model previously (Ucisik et al., 2013a). Moreover, cationic liposomes interact with serum proteins, lipoproteins, and the extracellular matrix of tissues, leading to the aggregation or release of the therapeutic agents before reaching the target cells and thus potentially causing systemic toxicity (Lv et al., 2006). Regarding the risk factors beyond the effectiveness, CurcuEmulsome, and PiperineEmulsome formulations were prepared as negatively-charged nanocarriers (Figure 2) (Fröhlich, 2012).

All emulsome formulations displayed a negative zeta potential, where CurcuEmulsomes distinguished with a higher negative charge, i.e., −34.23 ± 4.34 mV, which is further attributed to the contribution of molecular negative charge of curcumin to the formulation. The reverse correlation between zeta potential and the particle size is noteworthy (Du Plessis et al., 1996). CurcuEmulsomes differ from blank emulsomes and PiperineEmulsome with their slightly smaller average size. Based on the correlation between size and zeta potential, one can deduce that the higher negative zeta potential of CurcuEmulsome is the cause for their smaller average particle diameters with respect to blank emulsomes and PiperineEmulsomes that possess lower negative zeta potentials and are slightly larger in size (Table 1). In addition, the polydispersity index data shows a linearity between negative zeta potential and monodispersity of the formulations. CurcuEmulsomes and PiperineEmulsomes largely display monodispersed characters with PDI values close to 0.2 and 0.25, respectively.

It is also important to note that CurcuEmulsomes’ and blank emulsomes’ average diameters – i.e., 184.21 ± 13.30 and 239.12 ± 51.69 nm, respectively – were found to be smaller than those reported in our previous study, i.e., 286 ± 20.7 and 297 ± 28 nm, respectively (Ucisik et al., 2013b). In our view, shrinkage in particle sizes may be attributed to both/either (i) the presence of the sonication bath replacing the sequential extrusion steps applied in previous studies (Ucisik et al., 2013a, b, 2015a), and/or (ii) the lack of hexadecylamine that was present in the lipid composition of the previous studies (Ucisik et al., 2013a, b, 2015a). Hexadecylamine, an aliphatic amine molecule, was added previously to confer the emulsomes. A net positive surface charge that facilitates the recrystallization of S-layer proteins on the phospholipid surfaces for further surface functionalization purposes (Mader et al., 1999; Ucisik et al., 2013a). Since a positive surface charge is undesired in our study, hexadecylamine was excluded at our emulsome compositions.

#### Encapsulation

As previously described at Ucisik et al. (2013b), CurcuEmulsomes achieve curcumin encapsulation up to 0.10 mg/ml with an average of 0.07 ± 0.02 mg/ml, i.e., equivalent to 0.19 mM, throughout the study. Similar to curcumin encapsulation, highest piperine encapsulation was recorded as 0.09 mg/ml with an average of around 0,051 ± 0,02 mg/ml, i.e., equivalent to 0.18 mM. Interestingly in units of molar concentrations (i.e., in mM) very similar encapsulation levels were achieved for both curcumin and piperine. In other words, emulsomes are shown to be robust nanocarriers providing the same loading capacities for two different polyphenols in molecular level. According to the literature, when administered alone curcumin and piperine may induce a therapeutic effect at concentration ranges of 5–50 μM (Johnson and Mukhtar, 2007; Milacic et al., 2008; Hesari et al., 2019; Wong et al., 2019) and 10–150 μM (Lai et al., 2012; Yaffe et al., 2013; Greenshields et al., 2015), respectively. Curcumin and piperine concentrations within emulsomes are sufficient to achieve effective concentrations within the cell and therewith to display a therapeutic effect.

The encapsulation efficiency remained low, as the lipophilic compounds were added in excess amounts to the formulation. The efficiencies could be increased by decreasing the added amount of curcumin and piperine to the formulation without changing any other parameter.

#### Drug Release

The release profiles indicated release of nearly 40% curcumin and 7.5% piperine from their emulsome formulations (Figure 4). The low release profiles may be attributed to the solid character of emulsomes’ inner core. On the other hand, low stability of both compounds in water may have influenced the data of the analysis. For instance, curcumin is reported to own a half-life time less than 4.6 h in water at pH 7.3 (Tønnesen and Karlsen, 1985). On the other hand, the *cis–trans* isomerization of piperine in isopiperine, isochavicine and chavicine by UV-light has been reported (Ternes and Krause, 2002; Kozukue et al., 2007). The existence of three isomers (Ternes and Krause, 2002; Kozukue et al., 2007) – i.e., chavicine, isochavicine, and isopiperine – was monitored on HPLC analysis (Figure 5), indicating conversion of piperine to its isomers at certain extent as the contact of the sample with light was not entirely evitable during and after production of PiperineEmulsomes. Therefore, it is important to state that the release studies did not take into account the amount of piperine isomers. The amount of piperine released by PiperineEmulsomes might have been likely underestimated due to light-induced isomerization. Furthermore, the slow release profiles of both CurcuEmulsome and PiperineEmulsome may be attributed to the absence of enzymatic degradation of the solid lipid composition under *in vitro* conditions (Garcia-Fuentes et al., 2005). Yet, in cell following the uptake, emulsomes are known to accumulate inside the endosomes (Ucisik et al., 2013a, b), and release is expected to occur via enzymatic degradation, and in higher extent than during the *in vitro* release profile.

### Cell Culture Studies

In the present study, the effect of curcumin, piperine Curcuemulsome, PiperineEmulsome and their combination were examined *in vitro* on HCT116 colon cancer cell model for better understanding of cell cycle, apoptosis and gene expression induced by curcumin. Studies indicate that curcumin is a potent inhibitor of growth in colon cancer cells (Goel et al., 2001) and 35 μM curcumin treatment optimally induces apoptosis in HCT116 CRC cells (Collett and Campbell, 2004). Piperine-mediated inhibition of colon cancer was detected at 75–150 μM showing a cytostatic effect and causing G1 phase cell cycle arrest (Yaffe et al., 2015).

#### Cell Viability

Parallel to these findings, in our study the cell viability of HCT116 cells was significantly decreased after treatment of curcumin. Although, piperine did not affect cell viability of HCT116 cells, treatment of combination of curcumin and piperine decreased cell viability of HCT116 cells more than curcumin alone, suggesting an additive effect of piperine. This combination therapy led to a dose and time dependent decrease of cell viability of HCT116 cells. 25 μM CurcuEmulsome and 7 μM PiperineEmulsome concentrations were found as optimum to achieve highest therapeutic effect on HCT116 cells (Figure 6), and thus were selected to study the anti-cancer effects of the therapy further on apoptosis, cell cycle arrest and caspase 3 gene expression.

Both free curcumin and CurcuEmulsome treatment resulted in a gradual decrease in viability of HCT116 cells while the concentration increases from 5 to 50 μM (Figure 6). Yet, free piperine and PiperineEmulsomes caused no significant change in viability of HCT116 cells. Indicating the contribution of piperine to curcumin’s biological activity, combination of 7 μM PiperineEmulsome with 25 μM CurcuEmulsome was found to have the highest inhibition on cell proliferation: cell viability of around 50% was observed at 48 and 72 h (Figure 6F). While its presence as the second drug agent reduced the cell viability, no inhibition of cell proliferation was observed for piperine or PiperineEmulsome alone within the concentration range tested. This behavior was previously reported by Tang et al. (2017) on drug-resistant human ovarian A2780/Taxol cells (Tang et al., 2017). While high concentrations are required for piperine to show its anticancer effect alone, when co-delivered with curcumin, low concentrations of piperine are sufficient to contribute curcumin’s anti-cancer effect (Shoba et al., 1998). For instance in the review of Prasad et al. (2014), 1/100 w/w ratio was recommended (Prasad et al., 2014). Likewise, Vecchione et al. (2016) applied piperine as 1/100 w/w as compared to curcumin amount, to study the effect of a chitosan-based curcumin-piperine formulation on HT29 colon cancer (Vecchione et al., 2016). Jalili-Nik et al. (2018) reported that 1/25 w/w ratio appears suitable to improve bioavailability and maintenance of curcumin in the body tissues. In our study with emulsome formulations, piperine/curcumin ratio varied between 1/1.3 and 1/46 w/w ratio. Very slight difference in MTS data was observed for the treatment groups having piperine/curcumin w/w ratios of 1/4.6 (i.e., 7 μM PE/25 μM CE), 1/11.6 (i.e., 2.8 μM/25 μM) and 1/46 (i.e., 0.7 μM PE/25 μM CE), while still the latter was seem to be slightly most effective treatment group and selected for the further apoptosis and cell cycle studies.

Patil et al. (2016) studied the interaction of piperine with curcumin on combined quantum chemical and molecular docking techniques and concluded that piperine (i) forms an intercalation complex with curcumin, which further aids in transport of curcumin, and (ii) inhibits certain cytochrome P450 family of monoamine oxidase enzymes such as CYP3A4, UDP-glucuronosyltransferase (UGT) and UDP-glucose dehydrogenase (UDP-GDH) that are responsible with glucuronosylation of curcumin (i.e., elimination from the body), thereby enhancing the bioavailability as well as biological activity of curcumin (Patil et al., 2016).

#### IC_50_ Value of CurcuEmulsomes

Using the cell viability data of 72 h, IC_50_ values were estimated as 11.08 ± 1.31 and 19.69 ± 3.27 μM for free curcumin and CurcuEmulsomes, respectively (Figure 7). Although the increase in IC_50_ value indicates fewer efficacies of the CurcuEmulsomes compared to free curcumin dissolved in DMSO, the increase in IC_50_ is not related with the efficacy, but the amount of curcumin actively present inside the cell. Although the same amount of curcumin was administered to the cell medium, in both free and emulsome formulations, the amount of curcumin released inside the cell is not the same based on two characteristics of the nanoformulation. Firstly, due to its size in range of 100–300 nm, CurcuEmulsomes’ cellular uptake precedes endocytosis, rather than diffusion as it occurs for free curcumin with its molecular size. Secondly, due to the solid state of the inner matrix, also after internalization it takes a further time until the total amount of curcumin has been released into the cytoplasm from the endosomes where emulsomes were shown to accumulate at least as long as 72 h following the endocytosis (Ucisik et al., 2013a, b). Therefore, the higher IC_50_ value of CurcuEmulsomes is attributed to the slower internalization time as well as prolonged release of curcumin inside the cytoplasm.

#### Cellular Uptake

Biological efficacies of CurcuEmulsomes and PiperineEmulsomes were studied both separately and combined (as a dual therapy approach) *in vitro* on HCT116 colon cancer cell model in order to further investigate the additive effect of two natural polyphenols, as well as to evaluate the potential of emulsomes in delivering the compounds to the cells. While studying the uptake of CurcuEmulsomes separately, the autofluorescence properties of curcumin (Ucisik et al., 2013b) allow tracing the cell uptake of CurcuEmulsomes. No overlap between the signals of DAPI and CurcuEmulsome was detected, indicating that emulsomes remain inside the cell membrane and within the cytoplasm, but do not penetrate into the nucleus. Parallel to the findings of Ucisik et al. (2013b), signals at certain spots within the cytoplasm were detected at 24 h to be particularly strong (Figure 8B) (Ucisik et al., 2013b). This observation confirmed once again that emulsomes’ uptake follows endocytosis and emulsomes are accumulated within the endosomes prior any release to the cytoplasm. Lipophilic membrane dye pKH26 labels both the cell membrane as well as CurcuEmulsomes owing a membrane-like phospholipid bilayers in its outermost shell due to the aliphatic tail of pKH26 dye that rapidly intercalates into exposed lipid bilayer (Wallace et al., 2008). Therefore, while curcumin inside the CurcuEmulsomes emits light in green spectrum, pKH26 inside the cell membrane and the outermost shell of CurcuEmulsomes emits light red. DAPI labels the nucleus. Depending on the strength of the fluorescence intensities, CurcuEmulsomes appear in green or yellow in the merged image. The red spots with strong fluorescence are affiliated to the assembled endosomes carrying the CurcuEmulsomes inside. CurcuEmulsomes were observed to be localized first on the cell membrane of HCT116 cells, then internalized via endocytosis inside the cytoplasm at 24 h (Figures 8B,C). This findings are parallel to our former study where hepatoblastoma-derived HepG2 cell line was treated with emulsomes and the occurrence of endocytosis was monitored by intrastructural transmission electron microscopy analysis of thin-sectioned cells after treatment (Ucisik et al., 2013a). The fluorescence intensity of CurcuEmulsomes is observed to be highest at 24 h, which qualitatively confirms the flow cytometry data revealing that 95.9% of the CurcuEmulsomes were internalized after 24 h of treatment (Figure 8A).

In Figure 9, the internalization of CurcuEmulsomes (25 μM) was observed in both absence and presence of PiperineEmulsomes (7 μM). Higher fluorescence intensity of CurcuEmulsomes in the presence of PiperineEmulsomes was attributed to occurrence of a higher uptake. These findings is parallel to the previous studies reporting that the extent of curcumin uptake is improved significantly by piperine addition (Gülseren et al., 2014; Tang et al., 2017). Likewise, Lund and Pantuso demonstrated that addition of piperine resulted in a 229% increase in permeability of curcumin across intact Caco-2 monolayers (Lund and Pantuso, 2014).

#### Cell Morphology

In line with the cell viability data, PiperineEmulsome treatment did not cause any change in the morphology of HCT116 cells (Figure 10B). Treatments with CurcuEmulsomes resulted in transformation of cells to round-shaped cell morphologies (Figure 10C) which is attributed to the loose of integrity and detachment of the HCT116 cells induced to apoptosis (Ucisik et al., 2013b; Yallapu et al., 2014). Observed morphological changes such as shrinkage, spherical shape and aggregation were clear signs of apoptosis (Lane et al., 2005). Besides, when PiperineEmulsome is co-delivered with CurcuEmulsomes, an increase was observed in the number of cells with lost integrity (Figure 10D). The increase in number of cells with lost integrity was further attributed to the contribution of piperine to biological effect of curcumin.

#### Cell Cycle Arrest

CurcuEmulsome treatment resulted in a cell cycle arrest in G2/M phase. HCT116 cell population in G2/M phase has increased from around 37.9 ± 6.8% to around 47.1 ± 7.4% after 24 h treatment (Figure 11A). Interestingly treatment of PiperineEmulsome showed no G2/M phase arrest, while a slight, insignificant increase was observed in G0/G1 phase cell population. When cells were treated with both emulsome formulations, G2/M cell cycle arrest was again observed compared to control group. The increase in G2/M phase cell population, i.e., from 37.9 ± 6.8 to 47.4 ± 10.2%, seemed to remain less than observed for CurcuEmulsome alone. Interestingly, while the cell population accumulated in G2/M phase upon treatment with CurcuEmulsomes – either alone or combined with PiperineEmulsomes – within the first 48 h, it decreased in numbers when treatment reaches to 72 h (Figure 11C). Together with the cell viability data one can deduce that the cells in G2/M cell cycle arrest have undergone apoptosis and the decrease in cell population at G2/M phase after 72 h is related with this cell death occurring following G2/M arrest. Apart from G2/M phase arrest, S phase arrest was observed for cells treated 72 h with CurcuEmulsomes, either alone or combined with PiperineEmulsome (Figure 11C). These findings are consistent with findings of Mosieniak et al. (2012), where an increase at cell population in S phase was observed in HCT16 cells when treated with more than 15 μM of curcumin (Mosieniak et al., 2012). In that study, S phase arrest was associated with the induced apoptosis to cells at G2/M arrest.

#### Apoptosis: Flow Cytometry Analysis and Caspase 3 Gene Expressions

After 48 h treatment with CurcuEmulsomes, 4.2 ± 0.6% of HCT116 cells were found in early apoptotic stage and 31.9 ± 11.7% of cells at late apoptosis, whereas those treated with combination of CurcuEmulsome and PiperineEmulsome were with 5.9 ± 5.8% at early apoptosis and 43.6 ± 5.1% at late apoptosis (Figure 12). The present study reveals an increase in apoptosis level when HCT116 cells treated with CurcuEmulsome and PiperineEmulsome combinations at 72 h. Treatment with combination of CurcuEmulsome and PiperineEmulsome led HCT116 cells with 2.3 ± 1.0% to early apoptosis and with 81.0 ± 9.1% to late apoptosis. These results suggest that CurcuEmulsomes are capable to induce apoptotic pathway and PiperineEmulsomes contributes to the level of apoptosis at 48 h. While at 72 h treatment of HCT116 cells with combination of CurcuEmulsome and PiperineEmulsome did not show a significant change compared to CurcuEmulsome treatment alone. This suggests either PiperineEmulsome increases the cellular uptake of CurcuEmulsome in HCT116 cells showing the anticancer effect before 72 h, or the combination therapy leads the HCT116 cells to programed cell necrosis faster indicating that curcumin inhibits in a time-dependent manner. These findings were verified at gene level where Caspase 3 level of the cells were increased by fourfold after CurcuEmulsome treatment. When the treatment is combined with PiperineEmulsomes, the increase was escalated to sixfold (Figure 12C) suggesting that PiperineEmulsomes enhances the anticancer activity of CurcuEmulsomes. Our findings are consistent with the previous studies in the literature reporting that curcumin triggers apoptosis in colon cancer cells by activating caspase 3 (Jaiswal et al., 2002; Rashmi et al., 2005; Su et al., 2006; Zhang et al., 2017; Jalili-Nik et al., 2018). Likewise in another study, curcumin therapy on the pancreatic cancer PANC-1 cells resulted in a twofold increase in Caspase 3 gene expression levels indicating once more that the anticancer activity of curcumin proceeds over activation of caspases (Lu et al., 2018). Curcumin is also responsible for directly inducing membrane and DNA degradation and the nuclear shrinkage of cell (Mortezaee et al., 2019). Thus, this further explains the occurring morphological changes of HCT116 cells when treated with combination therapy. Induction of apoptosis in various type of cancer cells was reported for curcumin to occur via mitochondrial and receptor-mediated apoptotic pathways, where caspases play key role (Philchenkov et al., 2004; Xue et al., 2017).

## Conclusion

The present study formulates curcumin and piperine into emulsomes to enhance their limited bioavailability, and thus to achieve combinational anti-cancer effect on *in vitro* colon cancer model. The results demonstrate that when used in combination with CurcuEmulsomes, PiperineEmulsomes additively contribute to the anti-cancer activity of curcumin on HCT116 cells. The overall effect of combined therapy was studied through analysis on cell morphology, cell viability, cellular uptake, apoptotic cell death and cell cycle, as well as caspase 3 gene expression levels to further provide evidence how the two active molecules interact with HCT116 cancer cells in molecular basis. Observed morphological changes such as shrinkage, spherical shape, and aggregation were clear signs of induced apoptosis. Cell cycle arrest at G2/M phase and the increase in Annexin V-positive cell population were further effects of induced apoptosis at combinational therapy. Sixfold elevated gene expression levels of Caspase 3 provided final evidence that PiperineEmulsome improves the anticancer effectiveness of CurcuEmulsomes.

Concisely, incorporation of curcumin and piperine into emulsomes results in water-soluble and stable drug delivery system that succeeds the delivery of curcumin and piperine into the cell. The enhanced *in vitro* anticancer efficacy of CurcuEmulsomes when combined with PiperineEmulsomes highlight the potential of the system for future *in vivo* and clinical studies. It is essential to emphasize that the present study is the first report disclosing emulsomes as the nanocarrier system achieving combinational cancer therapy with piperine and curcumin on HCT116 CRC cell line.

## Data Availability Statement

The raw data supporting the conclusions of this article will be made available by the authors, without undue reservation, to any qualified researcher.

## Author Contributions

ZI synthesized CurcuEmulsomes, performed the related characterization experiments, and calculated IC50 values for curcumin and CurcuEmulsomes. EY synthesized PiperineEmulsomes and performed the related characterization experiments. ZB executed and interpreted *in vitro* cell culture studies including cell proliferation, cellular uptake, cell cycle, Annexin V, and real-time PCR experiments. ZI and BD assisted ZB in cell culture experiments and analysis. MU designed the framework of the study, guided the conduct of studies, and together with FS supervised data analysis. FS provided resources for cell culture studies. ZB, ZI, and MU were actively involved in writing and revising the manuscript. All authors have read and approved the final manuscript.

## Conflict of Interest

The authors declare that the research was conducted in the absence of any commercial or financial relationships that could be construed as a potential conflict of interest. Based on the findings of this study, a patent application with file number 2019/05678 was recently accomplished to Turkish Patent and Trademark Office.
