# Supplementary material for: Curcumin- and Piperine-Loaded Emulsomes as Combinational Treatment Approach Enhance the Anticancer Activity of Curcumin on HCT116 Colorectal Cancer Model
Source: Front Bioeng Biotechnol. 2020 Feb 11;8:50. doi: 10.3389/fbioe.2020.00050 (PMC7026030; doi:10.3389/fbioe.2020.00050)
Supplement: Supplementary file 1 [file Table_1.DOCX]

Supplementary Material

Curcumin- and Piperine-Loaded Emulsomes as Combinational Treatment Approach Enhance the Anticancer Activity of Curcumin on HCT116 Colorectal Cancer Model

Zeynep Busra Bolat^1^, Zeynep Islek^1^, Bilun Nas Demir^1^, Elif Nur Yilmaz^2,3^, Fikrettin Sahin^1^, Mehmet Hikmet Ucisik^3,4*^

## Supplementary Figures

**Supplementary Figure 1.** NTA graph for (A) CurcuEmulsomes and (B) PiperineEmulsomes
